# Supplementary material for: From Clinical Specimen to Whole Genome Sequencing of A(H3N2) Influenza Viruses: A Fast and Reliable High-Throughput Protocol
Source: Vaccines (Basel). 2022 Aug 19;10(8):1359. doi: 10.3390/vaccines10081359 (PMC9412868; doi:10.3390/vaccines10081359)
Supplement: Supplementary file 1 [file vaccines-10-01359-s001.zip › vaccines-1725975-supplementary.pdf]

Table S1: Demographical and virological data of study population.

Sample ID	Gender	Age (year)	Province of origin	Sample type	Collection date	A(H3N2) strain name	A(H3N2) viral load (copies/ml)
**1**	Male	49,0	Milan	oropharyngeal swab	21 November 2016	A/Milano/77/2016	9,E+06
**2**	Male	12,1	Bergamo	oropharyngeal swab	22 November 2016	A/Milano/78/2016	4,E+07
**3**	Male	3,1	Bergamo	oropharyngeal swab	23 November 2016	A/Milano/79/2016	4,E+06
**4**	Male	4,2	Bergamo	oropharyngeal swab	05 December 2016	A/Milano/91/2016	2,E+07
**5**	Male	24,2	Brescia	oropharyngeal swab	15 December 2016	A/Milano/96/2016	2,E+06
**6**	Male	65,5	Milan	oropharyngeal swab	15 December 2016	A/Milano/98/2016	3,E+06
**7**	Male	13,9	Milan	oropharyngeal swab	19 December 2016	A/Milano/108/2016	4,E+07
**8**	Male	38,9	Mantova	oropharyngeal swab	19 December 2016	A/Milano/113/2016	2,E+06
**9**	Male	62,1	Varese	oropharyngeal swab	10 December 2016	A/Milano/100/2016	5,E+06
**10**	Male	69,7	Varese	oropharyngeal swab	20 December 2016	A/Milano/117/2016	2,E+06
**11**	Female	48,3	Mantova	oropharyngeal swab	12 December 2016	A/Milano/102/2016	6,E+04
**12**	Female	36,0	Bergamo	oropharyngeal swab	20 December 2016	A/Milano/118/2016	1,E+08
**13**	Female	14,7	Milan	oropharyngeal swab	27 December 2016	A/Milano/121/2016	2,E+06
**14**	Male	46,3	Milan	oropharyngeal swab	23 December 2016	A/Milano/119/2016	1,E+08
**15**	Female	5,2	Bergamo	oropharyngeal swab	28 December 2016	A/Milano/128/2016	8,E+07
**16**	Female	83,3	Varese	oropharyngeal swab	23 December 2016	A/Milano/120/2016	2,E+05
**17**	Female	78,3	Varese	oropharyngeal swab	27 December 2016	A/Milano/132/2016	5,E+06
**18**	Female	82,3	Varese	oropharyngeal swab	27 December 2016	A/Milano/133/2016	1,E+04
**19**	Female	50,4	Brescia	oropharyngeal swab	28 December 2016	A/Milano/146/2016	7,E+05
**20**	Female	59,0	Milan	oropharyngeal swab	15 December 2016	A/Milano/135/2016	4,E+06
**21**	Male	72,6	Mantova	oropharyngeal swab	30 December 2016	A/Milano/151/2016	1,E+08
**22**	Female	77,6	Mantova	oropharyngeal swab	29 December 2016	A/Milano/152/2016	7,E+08
**23**	Male	72,8	Mantova	oropharyngeal swab	27 December 2016	A/Milano/153/2016	1,E+06
**24**	Female	65,8	Brescia	broncho-alveolar lavage	03 November 2016	A/Milano/157/2016	9,E+07
**25**	Male	74,7	Brescia	broncho-alveolar lavage	05 December 2016	A/Milano/158/2016	3,E+08
**26**	Female	43,1	Milan	oropharyngeal swab	09 January 2017	A/Milano/67/2017	4,E+06
**27**	Male	50,7	Brescia	oropharyngeal swab	02 January 2017	A/Milano/3/2017	1,E+05
**28**	Male	67,8	Brescia	oropharyngeal swab	02 January 2017	A/Milano/4/2017	3,E+07
**29**	Female	71,5	Brescia	oropharyngeal swab	02 January 2017	A/Milano/5/2017	5,E+05
**30**	Male	61,6	Varese	oropharyngeal swab	02 January 2017	A/Milano/10/2017	3,E+07
**31**	Male	50,2	Milan	oropharyngeal swab	02 January 2017	A/Milano/17/2017	2,E+06
**32**	Male	84,2	Mantova	oropharyngeal swab	02 January 2017	A/Milano/19/2017	4,E+04
**33**	Male	78,8	Mantova	oropharyngeal swab	02 January 2017	A/Milano/21/2017	4,E+06
**34**	Male	14,5	Bergamo	oropharyngeal swab	10 January 2017	A/Milano/29/2017	2,E+05
**35**	Female	72,0	Milan	oropharyngeal swab	09 January 2017	A/Milano/31/2017	8,E+05
**36**	Male	74,7	Milan	oropharyngeal swab	10 January 2017	A/Milano/39/2017	3,E+08
**37**	Female	58,1	Brescia	oropharyngeal swab	04 January 2017	A/Milano/26/2017	2,E+07
**38**	Female	62,7	Brescia	oropharyngeal swab	05 January 2017	A/Milano/27/2017	2,E+06
**39**	Female	28,0	Brescia	oropharyngeal swab	10 January 2017	A/Milano/52/2017	8,E+07
**40**	Male	68,6	Brescia	broncho-alveolar lavage	07 January 2017	A/Milano/50/2017	4,E+06
**41**	Male	1,6	Brescia	broncho-alveolar lavage	03 January 2017	A/Milano/51/2017	4,E+07
**42**	Male	10,2	Bergamo	oropharyngeal swab	13 January 2017	A/Milano/57/2017	1,E+08
**43**	Male	6,3	Bergamo	oropharyngeal swab	13 January 2017	A/Milano/58/2017	1,E+10
**44**	Female	6,4	Bergamo	oropharyngeal swab	18 January 2017	A/Milano/71/2017	1,E+05

**45**	Female	76,0	Bergamo	oropharyngeal swab	17 January 2017	A/Milano/74/2017	4,E+05
**46**	Female	57,0	Bergamo	oropharyngeal swab	17 January 2017	A/Milano/77/2017	7,E+08
**47**	Male	62,0	Milan	oropharyngeal swab	12 January 2017	A/Milano/64/2017	6,E+05
**48**	Female	5,5	Milan	oropharyngeal swab	16 January 2017	A/Milano/80/2017	9,E+06
**49**	Male	72,2	Milan	oropharyngeal swab	16 January 2017	A/Milano/81/2017	1,E+09
**50**	Female	70,3	Milan	oropharyngeal swab	17 January 2017	A/Milano/82/2017	8,E+04
**51**	Female	84,3	Mantova	oropharyngeal swab	17 January 2017	A/Milano/85/2017	1,E+10
**52**	Male	80,6	Varese	oropharyngeal swab	16 January 2017	A/Milano/88/2017	2,E+06
**53**	Female	77,0	Brescia	oropharyngeal swab	18 January 2017	A/Milano/90/2017	4,E+06
**54**	Male	10,9	Bergamo	oropharyngeal swab	23 January 2017	A/Milano/103/2017	4,E+06
**55**	Female	72,9	Milan	oropharyngeal swab	23 January 2017	A/Milano/111/2017	3,E+09
**56**	Male	42,7	Mantova	oropharyngeal swab	25 January 2017	A/Milano/115/2017	4,E+07
**57**	Male	66,8	Mantova	oropharyngeal swab	19 January 2017	A/Milano/97/2017	1,E+05
**58**	Male	14,1	Brescia	oropharyngeal swab	24 January 2017	A/Milano/116/2017	1,E+08
**59**	Female	11,7	Brescia	oropharyngeal swab	23 January 2017	A/Milano/117/2017	2,E+07
**60**	Male	3,7	Brescia	oropharyngeal swab	19 January 2017	A/Milano/101/2017	1,E+07
**61**	Male	82,1	Brescia	broncho-alveolar lavage	24 January 2017	A/Milano/118/2017	2,E+07
**62**	Male	60,2	Brescia	oropharyngeal swab	26 January 2017	A/Milano/122/2017	4,E+06
**63**	Female	51,9	Brescia	oropharyngeal swab	30 January 2017	A/Milano/128/2017	9,E+04
**64**	Female	12,9	Bergamo	oropharyngeal swab	31 January 2017	A/Milano/132/2017	7,E+05
**65**	Female	41,1	Varese	oropharyngeal swab	27 January 2017	A/Milano/125/2017	9,E+05
**66**	Female	58,2	Varese	oropharyngeal swab	26 January 2017	A/Milano/126/2017	5,E+05
**67**	Female	3,0	Milan	oropharyngeal swab	30 January 2017	A/Milano/133/2017	9,E+05
**68**	Female	45,7	Milan	oropharyngeal swab	31 January 2017	A/Milano/137/2017	1,E+06
**69**	Male	10,0	Bergamo	oropharyngeal swab	07 February 2017	A/Milano/150/2017	1,E+05
**70**	Male	39,0	Brescia	broncho-alveolar lavage	30 January 2017	A/Milano/145/2017	2,E+05
**71**	Female	62,0	Brescia	broncho-alveolar lavage	02 February 2017	A/Milano/146/2017	4,E+05
**72**	Female	75,7	Brescia	broncho-alveolar lavage	02 February 2017	A/Milano/147/2017	3,E+09
**73**	Male	0,9	Brescia	oropharyngeal swab	08 February 2017	A/Milano/154/2017	6,E+05
**74**	Female	73,0	Mantova	oropharyngeal swab	10 February 2017	A/Milano/156/2017	6,E+07
**75**	Female	13,9	Mantova	oropharyngeal swab	10 February 2017	A/Milano/157/2017	3,E+08
**76**	Female	43,1	Brescia	oropharyngeal swab	16 February 2017	A/Milano/165/2017	1,E+07
**77**	Female	20,2	Monza-Brianza	broncho-alveolar lavage	02 December 2016	A/Milano/179/2017	1,E+06
**78**	Male	80,5	Milan	broncho-alveolar lavage	04 January 2017	A/Milano/181/2017	2,E+07
**79**	Male	82,0	Monza-Brianza	broncho-alveolar lavage	11 February 2017	A/Milano/184/2017	3,E+05
**80**	Female	70,4	Monza-Brianza	broncho-alveolar lavage	17 January 2017	A/Milano/182/2017	2,E+07
